# Provider perceptions of lack of supportive care during childbirth: A mixed methods study in Kenya

**DOI:** 10.1080/07399332.2021.1961776

**Published:** 2021-09-17

**Authors:** Laura Buback, Joyceline Kinyua, Beryl Akinyi, Dilys Walker, Patience A. Afulani

**Affiliations:** a UCSF Institute for Global Health Sciences, San Francisco, California, USA;; b Kenya Medical Research Institute, Nairobi, Kenya;; c Global Programs for Research and Training, Kenya, Nairobi, Kenya;; d School of Medicine, University of California, San Francisco (UCSF), San Francisco, California, USA

## Abstract

Supportive care is a key component of person-centered maternity care (PCMC), and includes aspects such as timely and attentive care, pain control, and the health facility environment. Yet, few researchers have explored the degree of supportive care delivered or providers’ perceptions on supportive care practices during childbirth. The researchers’ aim is to evaluate the extent of supportive care provided to women during childbirth and to identify the drivers behind the lack of supportive care from the perspective of maternity providers in a rural county in Western Kenya. Data are from a mixed-methods study in Migori County in Western Kenya with 49 maternity providers (32 clinical and 17 non-clinical). Providers were asked structured questions on various aspects of supportive care followed by open ended questions on why certain practices were performed or not. We conducted descriptive analysis of the quantitative data and thematic analysis of the qualitative data. We analyzed data and found inconsistent and suboptimal practices with regards to supportive care. Some providers reported long patient wait times in their facilities as well as the inability to provide the best care due to staff shortages in their facilities. Others also reported low interest and inquiry about women’s experience of pain during childbirth, which was driven by perceptions of pain during childbirth as normal, facility culture and norms, and lack of pain medicine. For the facility environment, providers reported relatively clean facilities. They, however, noted inconsistent water and electricity as well as inadequate safety. We conclude that many drivers of the lack of supportive care are caused by structural health systems issues, therefore a health system strengthening approach can be useful for improving the supportive care dimension of PCMC, and thus quality of care overall.

Maternal mortality remains a significant public health burden in developing countries, with sub-Saharan Africa (SSA) accounting for about two-thirds of the global burden of maternal deaths (Alkema et al., 2016). Yet maternal mortality is not a public health issue alone; high maternal mortality is a threat to advancing human rights, education, and equity for all practitioners in international health & development. To advance the status of women worldwide, we need to understand the barriers to reducing maternal mortality, which includes poor quality of maternal healthcare. Poor quality of care contributes significantly to maternal deaths (Miller et al., 2016). Recently, the World Health Organization (WHO) and other international bodies on human reproduction programs have emphasized the need for more research to better understand poor maternal care during facility-based childbirth, especially in rural settings (Downe, 2019; WHO, 2007). While several researchers have documented the lack of supportive care from the perspective of women, we have much to learn about the drivers of this unsatisfactory care from the perspective of maternity care providers (Downe, 2019). Our aim in this study is to evaluate the extent of supportive care provided to women during childbirth and to identify the drivers of the lack of supportive care from the perspective of maternity providers. Through this study in Kenya, we reveal experiences of women during childbirth, as well as perspectives of maternity providers, that may be similar in other low- and middle-income countries (LMICs).

## Background

According to the WHO Quality of Care Framework for Maternal and Newborn Health (2015), quality of care includes dimensions of both provision of care and experience of care (Tunçalp et al., 2015). Experience of care includes three elements: effective communication, respect and dignity, and emotional support. We use the person-centered maternity care (PCMC) framework in this paper. The PCMC framework emphasizes women’s experience of care, and is described as maternity care that is respectful and responsive to the needs and preferences of women (Anonymous et al., 2017; Downe, 2019). The three PCMC domains ensure a good relationship between the woman and provider which builds trust and promotes positive perceptions of the healthcare encounter (Sudhinaraset et al., 2020). Poor PCMC is a violation of women’s human rights and is associated with adverse pregnancy outcomes. In this paper, we focus on the supportive care domain of PCMC, which includes components such as timely and attentive care, pain control, social and emotional support, and the health facility environment (Afulani et al., 2017.

Timeliness emphasizes the third of the three delays that lead to maternal mortality—the delay to receive adequate care after a woman arrives at the facility—which follows the first two delays of seeking care and arrival at the health facility (Thaddeus & Maine, 1994). Other researchers have shown that this third delay accounts for a significant proportion of maternal deaths. Timeliness and attentive care are critical to ensuring that complications are identified and managed quickly to prevent severe morbidity and mortality. Women also expect attention and compassionate care from their providers and the absence of these lead to negative experiences, which affects the decision to seek care in the future (Srivastava et al., 2015). Furthermore, international guidelines recommend women have choices on pain control depending on their preferences, ranging from relaxation and manual techniques to epidural analgesia and parenteral opioids (WHO, 2018). However, in prior studies, researchers have documented women’s experiences of neglect and abandonment during childbirth, often resulting in women delivering alone without skilled attendants within health facilities, and without pain control (Balde et al., 2017; Bohren et al., 2015; D’Ambruoso et al., 2005).

In this study, we delve into the drivers of the lack of supportive care from the perspective of maternity providers using both quantitative and qualitative data from Western Kenya.

## Methods

The data in this paper are from a larger mixed-methods study on community perceptions of quality of maternity care in a rural county in Western Kenya, which we have described in detail elsewhere (Afulani et al., 2020a; Afulani et al., 2020b). Migori County has eight sub-counties, each of which has a sub-county hospital in addition to several health centers. The county has 32 nurses, 19 clinical officers, and four doctors per 100,000 people (Kenya National Bureau of Statistics, Ministry of Health, National AIDS Control Council, Kenya Medical Research Institute, National Council for Population and Development, Nairobi, Kenya, and The DHS Program, ICF International, Rockville, Maryland, USA, 2015). It has a population of about one million with an estimated 40,000 births annually (Migori County Department, 2016). Maternal and child health indicators are generally lower in this county than in other counties in Kenya with an estimated maternal mortality ratio of 673 deaths per 100,000 live births compared to the national average of 495; approximately 53% of births in the county occur in health facilities, compared to the national average of 61% (Kenya National Bureau of Statistics, Ministry of Health, National AIDS Control Council, Kenya Medical Research Institute, National Council for Population and Development, Nairobi, Kenya, and The DHS Program, ICF International, Rockville, Maryland, USA, 2015).

We obtained the data for this paper from forty-nine clinical and non-clinical providers working in maternity units across all sub-counties of Migori County. Providers were purposefully sampled from 18 facilities selected for an intrapartum quality improvement project based on their relatively higher volume of births and we conducted interviews in October and November 2016. The 18 facilities were spread across all eight sub-counties of Migori County. In this study, we employed a convergent mixed-methods design in which both quantitative and qualitative date were obtained at the same time. Two female research assistants conducted the interviews using a questionnaire with both closed and open-ended questions. The interviews were conducted in English, Swahili or Luo in private spaces in each health facility. Each interview lasted about an hour. The structured responses were directly entered into the REDCap application (Harris et al., 2009). Additionally, the interviews were audio-recorded and transcribed (with simultaneous translation where necessary). All participants provided written informed consent. The study was approved by the ethical review units of the author’s institutions.

We operationalized supportive care by using items in the supportive care sub-scale of the PCMC scale, which captures the WHO minimum standards (Afulani et al., 2017). The key components of supportive care we explore in this paper are timely and attentive care, pain control, and the environment. Although birth companionship is considered part of supportive care, it is not included in this paper as we have discussed it previously (Afulani et al., 2018). We have also written other papers where we have examined the dignity and respect and communication and autonomy domains (Afulani et al., 2020a; Afulani et al., 2020b). Providers were asked to assess the relative frequency at which certain practices related to supportive care occurred using closed-ended questions with structured responses. They were then asked open-ended follow-up questions regarding their responses to the structured questions in order to assess why certain behaviors were practiced or not practiced and why. While we focused questions on childbirth, our open-ended question approach often led to providers to discuss other components of care as well. For example, when discussing timeliness of care, many providers also discussed the situation of antenatal care (ANC).

### Data analysis

We conducted both quantitative and qualitative analysis for the study. We used descriptive statistics to examine the characteristics of the providers and their responses to the structured questions on supportive care and bivariate analyses to examine the associations between reporting on supportive care and various demographics. We then analyzed the qualitative data to identify themes using the approach described by Braun and Clarke (2006). We generated themes both inductively and deductively using an initial codebook based on the questions and codes generated from open coding 10 transcripts. This codebook was used by the rest of the team (four coders) to code the rest of the transcripts and the codebook was continuously updated to incorporate new emerging codes from the remaining transcripts. We wrote analytic and reflexive memos to capture reactions to the data and emerging themes. We then iteratively analyzed the codes and coded text and reviewed our memos to generate categories and identify themes. We considered both the semantic (surface) and latent (underlying) meaning of the text and focused on salience (rather than frequency) for the qualitative data. We analyzed quantitative data with STATA 15 (StataCorp, 2017) and qualitative data were analyzed using Atlas.ti 8.4 (ATLAS.ti, 2016).

## Results

### Demographics

The characteristics of the 49 providers in the sample are shown in Table 1 and have been described in previous publications (Afulani et al, 2018; Afulani et al., 2020a; Afulani et al., 2020b). Thirty providers worked in public hospitals (county and sub-county hospitals), 13 in health centers, and six in mission/private hospitals. The respondents included seven clinical officers, 25 nurses and midwives, and 17 non-clinical staff (including cleaners and cooks). (See Appendix 1 for demographics by provider and facility type).

### Supportive care domains

We show providers’ assessment of the extent of various supportive care practices through quantitative results in Table 2 (See Appendix 2 for details by provider and facility type). We complement this by shedding light on the drivers of the lack of supportive care and facilitators of supportive care through the discussion of our qualitative findings.

### Timeliness and attentive care

Thirty-nine percent of providers reported women wait somewhat or very long in their health facilities to receive care. We found long wait times, however, only in the public hospitals and health centers, where providers reported this to be over 40%, compared to 0% for the mission/private hospitals. These wait times were often in reference to ANC rather than Labor and Delivery (L&D). For indicators of attentive care, providers in government hospitals were consistently lower than providers in health centers. About a third of all providers reported taking the best care of women all of the time (29%), though only 17% in government hospitals compared to 46% in health centers. A third of all providers also reported showing they cared all of the time (33%), which was similarly lower in government hospitals (21%) than health centers (46%). Likewise, one third of providers reported always paying attention to women during their stay (37%): this was 27% in government hospitals and 54% in health centers.

We found the drivers of the lack of supportive care included staffing, workflow in the maternity and other units, prioritization of patients, and discrimination.

#### Staffing

Most providers noted staff shortage is a key reason for women waiting long to be seen, not getting full attention, and not being satisfied with care. About three-quarters (74%) of providers reported never having enough staff—with clinical providers reporting a much higher level of shortage (91%) than non-clinical staff (41%). Providers felt that because of the high workload they were unable to spend sufficient time with patients.

When there is huge work load especially the number of health worker, like one [to] five women who want to deliver, so after finishing another one you go to another so the other one will have waited for long.

(C-13)

We have few doctors and maybe they were attending to other patients and the woman is ready, or some women come when they are very ready, so before the nurse examines her, the baby is out but the person who accompanied the woman runs to call the nurse.

(NC-14)

Staff shortages not only affect timeliness of care but also attention and support necessary for respectful care. This sometimes leads to women being left alone or in care of non-clinical staff. Providers noted that when one provider was running several units or occupied with other duties, women in other units ended up waiting long or feeling neglected. For example, sometimes one clinical staff will be assigned to cover the outpatient, antenatal and maternity wards at the same time. This not only increases wait time for all, but also results in women in the maternity ward to be left in the care of support staff, who have to call the nurse when the woman is in the second stage of labor. This sometimes leads to situations where women birth their baby before the doctor or nurse arrives.

Because of the few staff in this facility-like you find one nurse both at the maternity, ANC and outpatient- and so when she is attending to a pregnant mother then the rest in the other departments must wait for her in the lines.

(NC-8)

When a patient comes and is to be taken to maternity, the nurse follows her to the maternity and there is one who remains at outpatient to take care of the sick. When the nurse is away then one of us the cleaners at the maternity are left with them.

(NC-7)

Staff shortages were real as well as artificial. For example, some providers mentioned insufficient staff was sometimes due to other staff frequently absent because they were at meetings or trainings. Also, wait times were sometimes longer in the morning because doctors had to go for meetings first before coming to see patients.

Sometimes they wait when the nurses are few, mostly nurses are never here as they go to seminars and we remain with only one nurse who is at the maternity and this sometimes make the patients to wait.

(C-9)

Sometimes in the mornings the doctors go for meetings and so the first patients who come in the morning have to wait for them until when they come back.

(NC-5)

Providers who reported women were always seen in a timely manner mentioned that this was because their facilities had added more providers and there was a dedicated nurse for the maternity unit, which enabled them to provide more supportive care. Sufficient staffing is thus a facilitator of supportive care.

They don’t wait for now … they added more doctors, you know before he was one person in OPD, maternity, looking after post-natal mothers and he was everywhere alone and so this made them to wait for some time before being attended to.

(NC-14)

In maternity they do not wait because there is always a nurse and when patients come, they are seen immediately.

(NC-3)

#### Workflow and organization of maternity units

While many providers noted staff shortages, they also noted the organization and distribution of tasks contributed to the lack of supportive care. For example, some non-clinical providers stated that they were the ones who stayed on the wards at night, with clinical providers usually sleeping elsewhere. A woman arriving in labor must, therefore, first describe her problem to the watchman who will decide if they need to come to the maternity ward, and then the woman is presented to the non-clinical support staff, who then calls the nurse or doctor. Women being neglected appeared more likely at night when only one provider was on duty.

At night we stay in the maternity and the doctor sleeps in another room, while the watchman stays around here. So whenever he hears the sound of a motorbike he will stand and ask them what the problem is whether is an ordinary patient or maternity. If the patient is for the maternity he will direct the patient to a room where one of the casuals [non-clinical staff] sleeps. Once the mother has been handed over to her, she will immediately inform the doctor that there is a patient. As the mother walks in maternity you will be able to know by the way she presents herself whether she needs quick attention or not. The doctor will come immediately to do examination then informs us of the stage where the mother is then he will tell us to continue with the observation and incase of anything we call him.

(NC-9)

Maybe if it is at night and the nurse is alone, then the woman may push alone but the nurse will come and assist the remaining procedures.

(NC-15)

Sometimes staff engaging in discussions among themselves or attending to other issues, when they were supposed to be attending to patients, also contributed to long wait times. In addition, there seemed to be a barrier to providing timely care when providers were on call, yet not readily available.

I may say somewhat long may be due to us … a client has come and you are still handling another situation, the clients will tend to wait especially when you are one staff on duty or two and there is a problem you are sorting out and a client has come, you will be attending to this client while others will be waiting.

(C-4)

Sometimes one is on call and the patient comes during odd hours like at night or so early, the person may be called but could be still engaged with other things.

(NC-17)

#### Contribution of other units

Longer wait times were sometimes due to delays in other units. For example, some providers noted that the laboratories were slow and often caused women to spend additional time waiting at the health facilities for results. Women were also said to sometimes wait long at the pharmacy to receive medications.

Because I don’t see in real time where they normally take long, except those who are being sent to the laboratory especially those who are coming through ANC. Because they have to use the system and at times the system there is a problem with technology … then they have to wait. But for those ones who don’t go to the lab they don’t wait for long.

(C-1)

I think the place where they take most time is the lab, and the pharmacy taking drugs. During ANC it depends with the time they come, if they pass through the lab and the line is long then they will have to wait.

(C-4)

#### Prioritization of patients

As they often found themselves understaffed and overburdened, providers attempted to improve timeliness of care by triaging and prioritizing emergencies. This, however, sometimes caused other women to experience delays in receiving care and to feel neglected.

… you know we have emergency cases, as I have told you that occasionally you find that we have only one nurse, the women who come for the clinic are always asked to wait as we have an emergency at the clinic … [after] we have cleared with this, we attend to them and this is because of shortage just as I have said that we don’t have nurses in specific department but it is good that our clients understand our challenges.

(C-9)

It can happen when the nurse has an emergency like someone with PPH or Eclamptic, the nurse must pay more attention to her and maybe she is alone, so she must be attended to first [before] the nurse can go to the other women.

(NC-1)

#### Discrimination

We also found evidence that timely care and wait times were not experienced by everyone in the same way and were sometimes linked with discriminatory and preferential treatment. Many acknowledged that staff as well as relatives and friends of staff were often given preference, including being moved ahead of the queue and treated in nicer ways, such as given warm water to bathe after birth. This was also the case for wealthier or higher socio-economic status women. Non-clinical providers were more likely to reference providers treating other staff and their friends and relatives better.

Yes a few times [Laughs] it happens mostly at OPD, they attend to their relatives and friends faster than the rest and they also love money, once they see how you are dressed then they will attend to you faster.

(NC-9)

I have not seen that happen here for the past two years that I have worked here, apart from maybe when a nurse is pregnant and wants to give birth then she is looked after first and we must give her warm water for bathing.

(NC-8)

In addition, some providers mentioned treating women who came to the facilities with their partners before others as a way of encouraging women to come with their partners. This might have inadvertently resulted in longer wait times for those who had no partners or could not come with their partners for various reasons, that might also be related to social status.

That is a plan we have made to sensitize them to be coming. This is our own making that when you come with your husband you will be taken care earlier than the others. This is to encourage them come with their partners. But if all of them come with their partners they will have to queue.

(C-30)

### Pain control

Pain control practices were inconsistent: 81% reported providers rarely (never or a few times) cared about treating pain and 57% reported providers rarely do *everything they can* to control pain. But, this varied for clinical and non-clinical providers, with 50% of clinical providers reporting they think doctors/nurses rarely do everything for pain control compared to 24% of non-clinical providers.

We found that several inter-related factors influence the pain control practices at the health facilities. These include facility culture and norms, providers’ perceptions of pain control, assumptions about women’s reports of pain, availability of drugs and supplies, and knowledge and skill in approaches to pain relief.

#### Facility culture/norms and perceptions of pain

The majority of providers mentioned pain control was primarily done through non-pharmacological means, such as verbal encouragement, back rubs, and breathing exercises. Efforts to reduce pain was linked to the perception that pain is “normal,” “natural,” and the “right direction,” and does not need to be treated unless very severe. A few providers mentioned that in their facilities, practices were set in place for pain control during labor including providing oral pain medicine and injections.

Things like labor you can’t control the pain but we do as much as possible to … let’s say you involve the patient in like talking, exercise, things like that so that she can forget but you cannot control the pain but you can make the mother feel like she has somebody around.

(C-17)

We normally encourage then and we are telling them that you cannot give birth without experiencing those pains, so we encourage them…. When delivering we don’t do anything [Laughing] we only encourage them, maybe psychological we tell them that with those contractions then the labor is on the right direction.

(C-9)

Though less frequent, few providers noted certain training or skills that dictated the type of care they provided for pain control, remarking the practices of what is learned in school.

During labor like we were told in school that you give pain killer or you apply [rub] the back but postnatal now Panadol works best.

(C-10)

You can advise the client to ambulate. You can also give psychotherapy, you talk to the patient so that she can be aware of where the pain is coming from and you can advice the patient to relax or you can also administer analgesics like Panadol (Paracetamol).

(C-12)

Others, however, noted that not much was done to control pain during labor and admitted this to be a challenge:

With the labor pains how can you control it surely, [Laughs] it is a natural pain you can just give an analgesia but it will not relieve as such … when the companion is there, then you tell her that during contractions you can rub the back to relieve her from the pain.

(C-3)

I think that (pain control) is still a challenge anyway, we just let these women labor naturally and bear that pain until they give birth, management of pain at delivery is not really that aggressive.

(C-10)

Non-clinical providers had mixed perceptions of pain control. While some were aware of injections and tablets, others did not know of any medicine or injections available for pain control.

There is a drug they give as an injection or some tablets to swallow when mothers complain of pain.

(NC-11)

I have never heard that there is any medicine for controlling pain while a woman is in labor.

(NC-17)

#### Assumptions about women’s reports of pain

We discovered that some providers’ perceptions about patients’ behavior became a barrier to providing compassionate care. For example, some providers designated certain women as hard to understand or exaggerating pain. One non-clinical provider even compared women to “*sheep*,” that did not do as they are told. Some were also perceived to hide how they were feeling which made it difficult to identify if they were in pain, and led providers to assume the pain is under control.

Sometimes they say it is hard for you to determine if it is really severe pain, some say these women are exaggerating the way they are showing it is not the way they are having it so they just assume.

(C-14)

When the mother is perceiving, and the doctor is also perceiving that it is normal pain, then the doctor is left to decide that there is no need for pain killer.

(C-17)

#### Availability of medicines and supplies

Providers also stated that lack of pain medication was a key barrier to proper pain control. Most providers only had paracetamol for pain control, which is of limited use for labor pains. Providers noted that pain medications were sometimes out of stock or the medications available were not very helpful for reducing labor pains. (Of note, only the county hospital, the only facility with cesarean section capability, has capability for providing epidural anesthesia).

Usually the supply of pain killers is bad because most of the times they are out of stock.

(C-15)

The unavailability of the drugs because we can give somebody like Panadol and the pain still goes on and we do not have any other option.

(C-12)

### Facility environment

Almost all (96%) providers reported their facilities to be clean or very clean, though only 49% reported having water all of the time and 31% electricity all of the time. Only 27% reported their facility is always safe. Inadequate support staff and supplies and poor facility infrastructure (including lack of toilets, space, water, electricity, and physical barriers for safety) were identified as barriers to a clean and safe environment.

#### Inadequate support staff and supplies

Non-clinical providers highlighted that having inadequate cleaning and security staff contributed to the lack of a clean and safe environment, and recommended the need to employ more support staff.

Staffs for doing cleaning should be added as we are so few.

(NC-13)

We have a soldier at night, but during the day, a patient can leave without anyone knowing. So it is not secure during the day.

(NC-12)

In addition to human resources, lack of cleaning and maintenance supplies and protective clothing also prevented optimal cleanliness.

They should buy for us the machine for cutting grass … this is because whoever is cutting grass the moment he is done with one area, then grass grows where he began with and this makes the facility to be bushy…. The person doing cleaning does not have uniform and hand gloves.

(NC-16)

They should bring to us the washing machines for linen, gum boots for the casual workers to put on and uniforms to be provided.

(NC-7)

One non-clinical provider reported that women not maintaining cleanliness of facilities were a barrier, and there was a need to teach women how to keep the facility clean.

… The patients should be taught on how to keep the cleanliness of the facility, we have dust bins but the patients still throw food stuffs all over.

(NC-1)

#### Poor facility infrastructure

##### Insufficient toilets and bathrooms

Insufficient toilets and bathrooms were emphasized as a challenge for sanitation and clean environment. This included toilets shared by men and women as well as sick patients.

We only have one toilet that is used by all, maybe someone has cholera and is using the same toilet with a woman who has given birth, it is not good.

(NC-15)

More bathrooms should be added as we have only one for both the male and the female. Though the ladies at the maternity have their bathroom, but general ward, there is only one bathroom that is shared by all the patients both male and female.

(NC-5)

One non-clinical provider even described a situation where birthing mothers were exposed to wet conditions when it rained because of the nature of the maternity ward.

There two doors here at the maternity, when it is raining, water enters the room and if it is in the evening when am home, the mothers stay in that wet area until when am back the next day to clean it up.

(NC-2)

##### Inadequate space and crowded environments

Inadequate space and crowded environments also was noted by providers to pose a threat to quality environment. Reasons for crowding included influx of patients from other areas (including neighboring countries such as Tanzania), increased demand due to confidence in good care, and availability of free services. Providers noted the space is sometimes insufficient for women delivering.

Like in maternity we need a bigger room as women are many.

(NC-1)

They are always so many because of the good treatment they get and also the tests that are being done here.

(NC-7)

Some also noted that they needed to improvise with space challenges, though the example below highlights how some of the improvising might lead to women birthing on the floor:

… you manage the situation the way it comes, because if now they come four, you have now to improvise and now put the mattresses down and just conduct [the birth] on the floor. But you cannot keep this woman waiting, you just have to improvise and use what is necessary at that moment.

(C-10)

##### Inconsistent water and electricity

Inconsistent water and electricity were also stated to be barriers to adequate environment and care, as they are necessary for sanitation and safety. Many noted inconsistencies of both water and electricity. Electricity was not always available due to lack of generator, rationing, or not paying the bills.

Sometimes it [electricity] gets lost … we have generator but it is still not working, so when the lights are off we light the lamps.

(NC-5)

There is [electricity] but not all the time. You might find the mother is in the maternity and there is no electricity and that brings challenge.

(NC-9)

##### Availability of water

Availability of water was also noted as sometimes dependent on rain and some cited the need for boreholes to improve their water supply.

We have water and borehole though because of the high population at times it is not enough.

(NC-1)

When it is not raining and is [water] is finished in the tank then we do not have water.

(NC-15)

##### Physical barriers to safety

Physical barriers to safety, such as fencing, locks, windows, lighting also challenge the supportive care environment. Overall, facilities were noted as generally safe, as all providers agreed that there were no high risks such as babies being switched or stolen, but it was nevertheless noted there are thefts and threats to safety facing staff and patients. While the above insufficiencies were noted primarily by non-clinical staff, clinical providers also emphasized the safety concerns.

It is good for the gate to be repaired so that when we are on night duty we should not fear to be attacked. The issue of the gate makes this place not safe because you find even robbers coming in at times.

(NC-9)

Any attacker can come from any corner because the barbed wires are old and some are down so security wise it is not good.

(C-9)

## Discussion

From conducting this mixed methods study, we learned there are suboptimal practices in aspects of supportive care related to timely and attentive care, pain control, and physical environment. The barriers to timely and attentive care include shortage of clinical staff, workflow in the maternity and other units, prioritization of patients, and discrimination. In addition, facility culture and norms, providers’ perceptions of pain control, assumptions about women’s reports of pain, availability of drugs and supplies, and knowledge and skill in approaches to pain relief contributed to inadequate pain control for birthing women. The facility environment was impacted by inadequate support staff, lack of cleaning and maintenance supplies, and poor facility infrastructure including insufficient toilets and bathrooms, inadequate space, inconsistent water and electricity, and inadequate physical barriers for safety. We believe that addressing these barriers will enable providers to provide more supportive care in a supportive environment.

Our identification of staff shortages as a barrier to timely and supportive care is not surprising given the low provider-patient ratios in the setting: 32 nurses, 19 clinical officers, and four doctors, per 100,000 people. Staff shortage was identified as a key barrier to other aspects of PCMC in the same study (Afulani, et al., 2020a; Afulani, et al., 2020b). The multiple roles of providers, which distract the cycle of the clinic, has also been discussed by other authors in similar contexts (Bradley et al., 2015; Thu et al., 2015). Shortage of clinical providers results in non-clinical staff playing critical roles in the continuum of care, which has been described by researchers elsewhere in Kenya (Golub et al., 2020). Such skill mix exacerbates the already present organizational challenges contributing to a decline in quality of supportive care (Gerein et al., 2006). We also found that processes and inadequate coordination with other units, such as laboratories and pharmacies, impacted wait times. While the laboratory is a significant unit of care, the turnaround time for testing has been previously identified by researchers as a major contributor to non-timely care (Soffiati & Giavarina, 2010). To improve timeliness of care, the providers report prioritization of more pressing cases, which increases the wait time for others and may leave some women feeling discriminated against as they wait. We suggest that adequate staffing, as well as communication of the situation to waiting women, are needed to reduce the wait time as well as prevent women’s perceptions of being discriminated against.

Provider reports of little interest or concern for women’s experience of labor pain is consistent with findings of other researchers on providers’ attitudes toward labor pain (McCauley et al., 2017, 2018). Additionally, it validates women’s experiences of unsatisfactory pain relief elsewhere in sub-Saharan Africa (Agnes et al., 2015; Mselle et al., 2019). In order for peripheral and provincial/county level health facilities to meet international guidelines for pain control (WHO, 2018), we recommend provider training and sensitization to the available pharmacologic and non-pharmacologic techniques, as well as improved supply chain for pharmacologic pain control. Further research into these perceptions and practices at various levels would also be useful.

Finally, although providers report relatively clean facilities, water, electricity, and safety were reported as inadequate. Non-clinical providers especially expressed concerns about the environment, as they are responsible for maintaining these conditions. It is important to note the role of shortage of support staff in maintaining the facility environment, since these roles are usually not given sufficient attention in discussions about the healthcare workforce in low resource settings. The role of facility infrastructure and supplies is also critical, as they not only impede supportive environment, but influence PCMC and quality of care in general (Thu et al., 2015).

The prominence of themes related to human resources, infrastructure, and availability of supplies/medicines emphasizes the importance of these factors in the provision of supportive care. These factors are highlighted in the WHO Quality of Care Framework for Maternal and Newborn Health (Tunçalp et al., 2015), in which authors illustrate that competent and motivated human resources as well as essential physical resources are underlying cross-cutting building blocks for both service provision and experience of care elements. Our results also demonstrate the links between the six building blocks (service delivery, human resources, medicines and technologies, financing, leadership and governance, and information) of the WHO Health Systems Strengthening (HSS) Framework (WHO, 2007). Women experiencing care within weak health systems are more vulnerable to not receiving supportive care, and in turn having lower experiences of care. Therefore, we suggest approaching PCMC from a health systems strengthening lens is critical to improving the dimensions of supportive care, as well as other dimensions such as dignified and respectful care and communication and autonomy ( Afulani et al., 2018; Afulani et al., 2020a; Afulani et al., 2020b; Smith et al., 2020).

### Limitations

Social desirability bias is a primary limitation in this study, as providers may overestimate the extent of supportive care in their facilities. Additionally, we included support staff in the study because of prior work highlighting their role in women’s healthcare experiences. This is a potential limitation given they have less knowledge of clinical aspects of care such as pain control. At the same time, we view this as a strength of the study, as support staff may respond from the perspective of participant observers hence provide less socially desirable results. This is particularly apparent when discussing pain control: clinical provider reports of the availability of higher pain control may be due to their training and knowledge of what is expected. On the other hand, non-clinical provider reports describing much less pain medication may be a better reflection of what is done, as they are reporting what they see without an understanding of what is possible, effective, and available. The different provider types may also influence the salience of issues highlighted, as seen by non-clinical providers being more vocal about the environment which they tend to be responsible for. Finally, there are issues of generalizability given the sample was drawn from one rural county.

## Conclusions

We find that practices needed to ensure supportive care are not optimal. Factors contributing to lack of timely and attentive care, insufficient pain control, and inadequate physical environment include shortage of both clinical and support staff, workflow in the maternity and other units, discrimination, facility culture and norms, provider’s knowledge and assumptions of labor pain control, availability of drugs and supplies, and poor facility infrastructure. Domains of the WHO HSS Building Blocks that can be specifically targeted to influence supportive care are human resources, supplies, and leadership/governance. Efforts to prevent shortage of clinical staff are necessary to ensure providers can provide timely and attentive care. This should include addressing issues that lead to artificial shortages. In addition, facilities need to employ adequate numbers of support staff to ensure a clean and safe environment. It is also necessary to train the limited staff in pain control including both pharmacological and non-pharmacological approaches. Commodity and stock management for medicines and supplies, in combination with training, is also essential for providers to properly manage pain during childbirth. Leadership and management strengthening are similarly essential for tackling the system weaknesses between providers, ancillary services (laboratories and pharmacies) and non-clinical support staff. We conclude that this lack of supportive care being driven by structural health systems issues suggests the need for a health system strengthening approach to improve the supportive care and other dimensions of PCMC and quality of care overall. This link can be leveraged in advocacy, funding, and implementation for PCMC and quality of care. Further research is, however, also needed to develop evidence-based interventions to improve supportive care during childbirth, to improve healthcare of women overall globally.

## Figures and Tables

**Table 1.** Distribution of provider characteristics (*N* = 49).

	No.	%
Facility type		
Govt. Hospital	30	61.2
Govt. Health Center	13	26.5
Mission Hospital	6	12.2
Position		
Clinical officer	7	14.3
Nurse/Midwife	25	51
Support staff	17	34.6
Sex		
Male	14	28.6
Female	35	71.4
Age		
Less than 30 years	9	18.4
30 to 39 years	21	42.9
40 or more years	19	38.8
Married	39	83
Number of children		
0 to 2	15	31.9
3 or 4	21	44.7
5 or more	11	23.4
Highest education		
Less than College	17	34.7
College and above	32	65.3
From County		
No	20	40.8
Yes	29	59.2
Years as a provider		
0 to 5 years	18	36.7
6 to 10 years	13	26.5
More than 10 years	18	36.7

**Table 2.** Distribution of provider responses on items related to supportive care.

	No.	%
How do you feel about the amount of time women wait when they arrive at the facility?		
Very short or don’t wait	13	27.1
Somewhat short	16	33.3
Somewhat long	17	35.4
Very long	2	4.2
Do the doctors, nurses, and other staff at the facility show that they care about the women?		
Yes, a few times	2	4.2
Yes, most of the time	30	62.5
Yes, all the time	16	33.3
Do the doctors and nurses at the facility talk to women about how they are feeling?		
Yes, a few times	7	14.3
Yes, most of the time	27	55.1
Yes, all the time	15	30.6
Do the doctors, nurses or other staff at the facility try to understand women’s anxieties and fears?		
No, never	2	4.1
Yes, a few times	14	28.6
Yes, most of the time	21	42.9
Yes, all the time	12	24.5
When women need help, do you feel the doctors, nurses or other staff at the facility pay attention?		
Yes, a few times	5	10.2
Yes, most of the time	23	46.9
Yes, all the time	21	42.9
Do the doctors and nurses ask how much pain women are experiencing?		
No, never	8	17
Yes, a few times	11	23.4
Yes, most of the time	13	27.7
Yes, all the time	14	29.8
Don’t know	1	2.1
Do you think the doctors or nurses do everything they can to help control women’s pain?		
No, never	14	28.6
Yes, a few times	14	28.6
Yes, most of the time	15	30.6
Yes, all the time	4	8.2
Don’t know	2	4.1
Do you feel like the doctors or nurses care about treating pain during or after labor and delivery?		
No, never	20	48.8
Yes, a few times	13	31.7
Yes, most of the time	5	12.2
Don’t know	3	7.3
Do you feel the doctors and nurses pay attention to women during their stay in the facility?		
Yes, a few times	3	6.1
Yes, most of the time	28	57.1
Yes, all the time	18	36.7
Do you feel the doctors, nurses or other staff at the facility take the best care of women?		
No, never	1	2
Yes, a few times	2	4.1
Yes, most of the time	32	65.3
Yes, all the time	14	28.6
Do you feel women completely trust the doctors, nurses or other staff at the facility with regards to their care?		
No, never	1	2.1
Yes, a few times	5	10.6
Yes, most of the time	23	48.9
Yes, all the time	15	31.9
Don’t know	3	6.4
Do you think there are enough health staff in the facility to care for women?		
No, never	36	73.5
Yes, a few times	7	14.3
Yes, most of the time	4	8.2
Yes, all the time	2	4.1
Did you feel the health facility is crowded?		
No, never	14	28.6
Yes, a few times	6	12.2
Yes, most of the time	23	46.9
Yes, all the time	6	12.2
Would you say the facility is very clean, clean, dirty, or very dirty?		
Dirty	2	4.1
Clean	41	83.7
Very clean	6	12.2
Is there water in the facility?		
Yes, a few times	9	18.4
Yes, most of the time	16	32.7
Yes, all the time	24	49
Is there electricity in the facility?		
Yes, a few times	6	12.2
Yes, most of the time	28	57.1
Yes, all the time	15	30.6
Is the health facility safe?		
No, never	18	36.7
Yes, a few times	5	10.2
Yes, most of the time	12	24.5
Yes, all the time	13	26.5
Don’t know	1	2

## Data Availability

The data analyzed for the manuscript are available from the second author on reasonable request.

## Abbreviations:

ANC: Antenatal Care
HSS: health systems strengthening
L&D: Labor and Delivery
LMIC: Low and Middle Income Countries
NC: non-clinical
OPD: Outpatient department
PCMC: person centered maternity care
SSA: Sub-Saharan Africa
WHO: World Health Organization
